# How Lipid-Specific T Cells Become Effectors: The Differentiation of iNKT Subsets

**DOI:** 10.3389/fimmu.2018.01450

**Published:** 2018-06-26

**Authors:** Haiguang Wang, Kristin A. Hogquist

**Affiliations:** Department of Laboratory Medicine and Pathology, Center for Immunology, University of Minnesota, Minneapolis, MN, United States

**Keywords:** invariant natural killer T cell, subsets, development, lipid, thymus, mucosal-associated invariant T cell

## Abstract

In contrast to peptide-recognizing T cells, invariant natural killer T (iNKT) cells express a semi-invariant T cell receptor that specifically recognizes self- or foreign-lipids presented by CD1d molecules. There are three major functionally distinct effector states for iNKT cells. Owning to these innate-like effector states, iNKT cells have been implicated in early protective immunity against pathogens. Yet, growing evidence suggests that iNKT cells play a role in tissue homeostasis as well. In this review, we discuss current knowledge about the underlying mechanisms that regulate the effector states of iNKT subsets, with a highlight on the roles of a variety of transcription factors and describe how each subset influences different facets of thymus homeostasis.

## Introduction

Natural killer T cells (NKT) were named because they express T cell receptor (TCR)–CD3 complexes as well as the natural killer (NK) cell receptor NK1.1 (CD161) (1, 2). Later, research discovered that NKT cells express a semi-invariant TCR, characterized by a Vα14-Jα18 TCRα chain coupled with a limited Vβ repertoire (Vβ2, Vβ7, or Vβ8.2) in mice, and an invariant Vα24-Jα18 paired with Vβ11 in humans (3, 4). Owning to this semi-invariant TCR, invariant natural killer T (iNKT) cells recognize self- and foreign-lipid antigens presented by the CD1d molecule and could be specifically detected using CD1d tetramer loaded with a cognate lipid antigen.

iNKT cells originate in the thymus, but in contrast to the conventional peptide specific CD4^+^ or CD8^+^ T cells, which are positively selected by cortical thymic epithelial cells, the positive selection of iNKT cells solely relies on the interactions among cortical double-positive (DP) thymocytes (5–7). DP thymocytes expressing the rearranged Vα14-Jα18 TCR recognize high-affinity lipid antigens presented by CD1d molecules on neighboring DP thymocytes (4). iNKT cells highly express the transcription factor promyelocytic leukemia zinc finger protein PLZF (*zbtb46*), which is essential for their effector program (8, 9), for specifying the tissue-resident properties of iNKT cells, and for their ability to produce cytokines early after stimulation (8–10).

It has been realized that iNKT cells are a heterogenous population, and recent evidence from various groups suggest that there are three major functional iNKT subsets at steady state according to their expression of lineage-specific transcription factors and cytokine-producing potential. The three iNKT subsets are designated NKT1, NKT2, and NKT17, in analogy to the classical CD4 T helper lineages. NKT1 cells are PLZF^low^ T-bet^+^ and produce both IFN-γ and low amounts of IL-4 after stimulation. They express NK1.1 and other NK receptors and represent the subset that “NKT” cells were named after. NKT2 and NKT17 cells, in contrast, do not express NK1.1. NKT2 cells are PLZF^high^ and produce high amounts of IL-4 at steady state and after stimulation. NKT17 cells are PLZF^intermediate^ ROR-γt^+^ and produce IL-17 after stimulation (11). Through intra-thymic transfer and fetal thymic organ culture (FTOC), previous studies demonstrated that each iNKT subset (NKT1, NKT2, and NKT17) is terminally differentiated; i.e., do not give rise to other cell subsets (11–13). iNKT cells play diverse roles in immunity due in part to the existence of these three functional subsets. The subsets produce distinct cytokines and reside in distinct tissues. With accumulating knowledge regarding the biology of iNKT cells, in this review, we summarize recent advances in the development and differentiation of iNKT subsets, as well as their role in maintaining the immune homeostasis.

## The Development and Differentiation of iNKT Cells

### Initial Positive Selection

**Figure 1:**
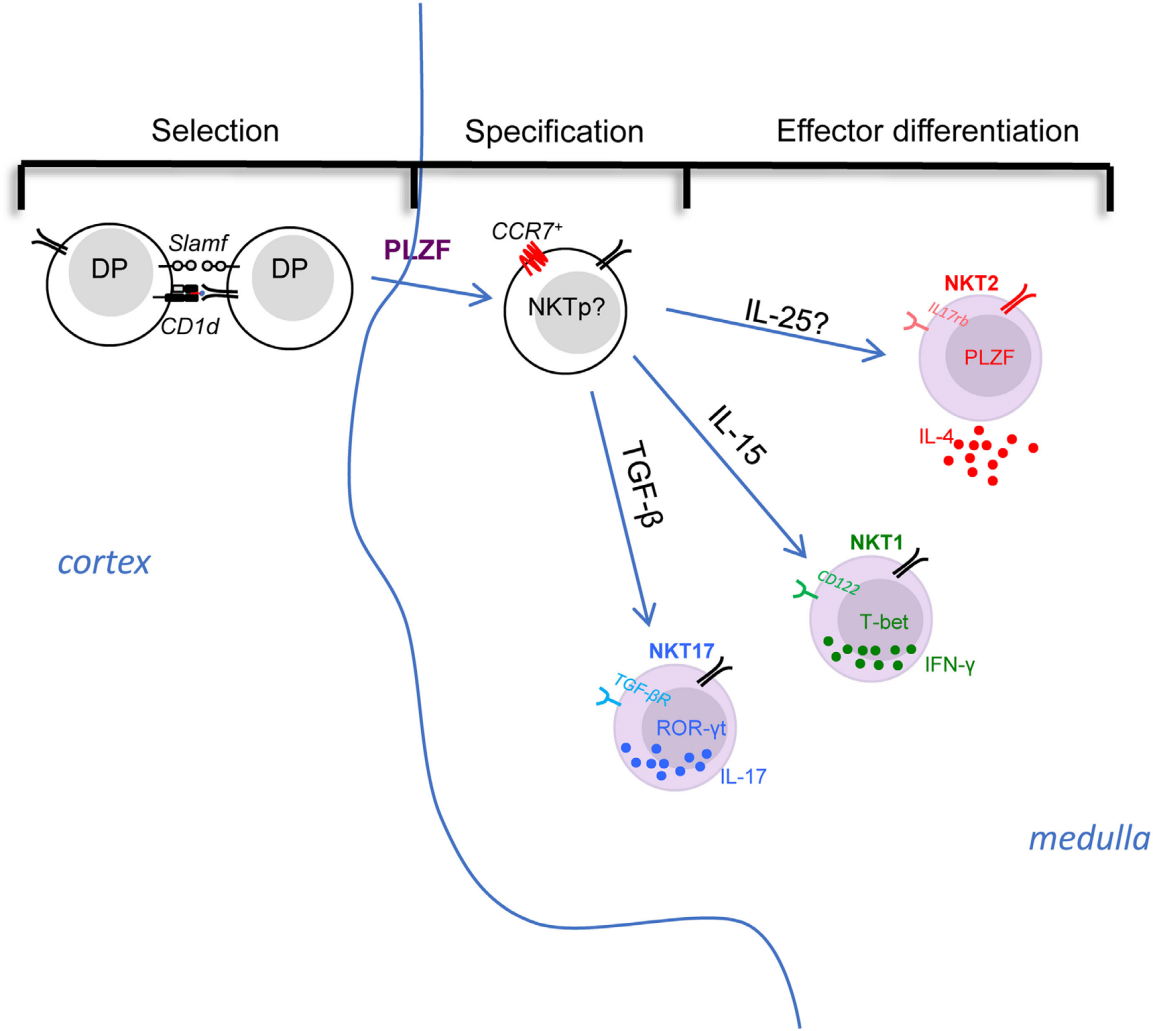
The selection, specification, and effector differentiation of invariant natural killer T (iNKT) cells in the thymus. The initial positive selection of iNKT cells depends on the interaction among double-positive thymocytes in cortex. Then, with the upregulation of PLZF and the chemokine receptor CCR7, the multi-potent iNKT common precursors migrate to thymic medulla and specify their fate into different effector subsets. The effector differentiation into NKT1, NKT2, and NKT17 are influenced and directed by various factors. For instance, cytokine signals play essential roles in differentiation: IL-15 is critical for CD122 expressing NKT1 cell differentiation, whereas TGF-β is required for TGF-βR-expressing NKT17 cells, while NKT2 cells express IL-17 RB (IL-25 receptor), although the role of IL-25 requires evaluation. iNKT subsets are poised in an effector state, but only NKT2 cells are actively producing cytokine (IL-4) at steady state, while NKT1 and NKT17 cells maintain potential to produce IFN-γ or IL-17, respectively, after stimulation.

Like the conventional CD4^+^ and CD8^+^ T cells, iNKT cells originate from precursors undergoing TCR gene rearrangement in the thymus. A lineage tracing study using transgenic mice (ROR-γt–Cre × ROSA26^lsl^-EGFP) in which ROR-γt triggers permanent expression of green fluorescent protein (GFP) confirmed that iNKT cells were derived from ROR-γt^+^ DP thymocytes in the thymus (5) (Figure 1 “Selection”), while a minor population could arise from DN thymocytes bypassing DP stage (14). Moreover, ROR-γt itself is essential for iNKT cell generation, in that, it supports DP survival through regulating Bcl-xL expression, allowing for optimal rearrangement of Vα14-Jα18 TCR chains (15). Similarly, an E protein transcription factor, HEB, promotes survival of DP thymocytes through regulating both ROR-γt and Bcl-xL expression, which opens the window of time to allow distal Jα rearrangement (16). Downstream of the initial selection of DP thymocytes, c-Myc has been shown to control the maturation of iNKT cells (17, 18). Moreover, c-Myb has also been shown to play a central role in this process, as it supports long half-life of DP thymocytes to allow Vα14 to Jα rearrangement (19).

Immediate post-selection precursor iNKT cells are characterized as CD1d tetramer^+^ CD44^−^ CD24^+^ CD69^+^, termed as “stage 0” iNKT cells. The strong TCR signal during iNKT selection was directly demonstrated using a reporter mouse in which a GFP cassette was inserted in the Nur77 locus (an immediate-early gene upregulated by TCR stimulation), wherein the GFP level indicates the TCR signal strength (20). In these mice, stage 0 iNKT cells express a high level of GFP indicating they received strong TCR signal during selection (20). Beside this strong TCR signal, the development of iNKT cells also relies on a “second signal” generated through homotypic interactions between signaling lymphocyte activation molecule family (SLAMF) receptors, SLAMF1 and SLAMF2, expressed on the DP thymocytes (21). In addition to supporting a long half-life in DP thymocytes, c-Myb also promotes the expression of CD1d and SLAMFs, which are essential for positive selection of iNKT cells (19). Deficiency of c-Myb completely abrogates the generation of iNKT cells, as CD24^+^ stage 0 iNKT cells were NOT detected (19).

Historically, in B6 mice, the maturation of iNKT cells beyond stage 0 was described as a stepwise linear model from stage 1 to 3 based on expression of CD44 and NK1.1. In this model, the stage 0 iNKT cells develop into CD24^−^ CD44^−^ NK1.1^−^ stage 1 cells, then upregulate CD44 to become stage 2 cells, and finally acquire NK1.1 expression to become stage 3 cells in a linear fashion (22). This model fits some but not all the available data. For example, NKT17 cells were known to express CD44 but not NK1.1 (stage 2), never become NK1.1^+^ (stage 3) (12). Alternatively, based on the expression of transcription factors, PLZF, Gata3, T-bet, and ROR-γt, CD24^−^ iNKT cells could be very well categorized into three distinct subsets, NKT1, NKT2, and NKT17 as described above. Similar to NKT17 cells, intra-thymic transfer of “stage 2” IL-4 producing NKT2 cells (IL-4^+^ IL-17RB^+^ CD4^+^) showed that they do not give rise to T-bet^+^ NK1.1^+^ “stage 3” cells either (11). Therefore, a revised lineage-diversification model for iNKT cell development, in which a common progenitor gives rise to the distinct lineages of NKT1, NKT2, and NKT17 cells (Figure 1) was suggested. We herein discuss the promoting and inhibitory factors for selection, specification, and differentiation of iNKT cells, which are summarized in Table 1.

**Table 1:** Genetic factors influence development of iNKT subsets.

Promoting factors	**Selection**	**Specification**	**Effector differentiation**		
			**NKT1**	**NKT2**	**NKT17**
	ROR-γt	PLZF	CD122/IL-15	IL-17RB/IL-25 ?	TGF-βRII/TGF-β

	HEB	Runx1	Let-7	Lin28	

	c-Myc	CUL3	Pak2		

	c-Myb	CCR7/CCL21a	Hobit	T cell receptor (TCR) binding half-life	

	SLAMF1 and SLAMF2	Egr2	UTX	KLF13	

	miR-181a	NKAP	T cell factor 1		
	TCR signaling strength	Histone deacetylase 3	Lymphoid enhancer factor 1 (preferentially promote NKT2)		
		Dicer/microRNAs			

Inhibitory factors			Lin28	Let-7	
	
			TCR binding half-life	Kruppel-like factor 2	TET2/TET3
	
				Jarid2	
				Ezh2	

### Specification

Stage 0 iNKT cells arise from DP thymocytes in the thymic cortex (6). However, in CD1d tetramer-based immunofluorescence and histocytometric analysis, thymic iNKT subsets were found to be predominantly localized in the thymic medulla (23) (Figure 1). Consistent with this, the thymic medullary environment was reported to impact the functional maturation of iNKT cells (24). Therefore, the nature and localization of the common progenitor that directly gives rise to distinct subsets is unclear. Furthermore, the signals that drive their migration from cortex to medulla, as well as the medullary factors that control the differentiation of iNKT subsets has not yet been reported. A previous study demonstrated that the chemokine receptor CCR7, which responds to the chemokines CCL21, is important for thymocytes trafficking from the cortex to the medulla (25). Additionally, the number of iNKT cells was significantly reduced in CCR7^−/−^ mice (26). Interestingly, single cell RNA-seq analysis of thymic iNKT cells suggested that PLZF^high^ iNKT cells might comprise a progenitor population (27). Previous work showed IL-4^−^ PLZF^high^ iNKT cells could further differentiate into T-bet^+^ NKT1 cells when sorted and intra-thymically transferred into thymus (11), suggesting they maintain precursor potential. Further analysis of this IL-4^−^ PLZF^hi^ iNKT cell population by RNA-seq and PCA analysis confirmed they have the least similarity to the three effector subset (28). Taken together, it could be inferred that CCR7^+^ cells within PLZF^high^ iNKT cells might serve as the common progenitor for iNKT subsets (Figure 1 “Specification”).

### Factors Involved in Specification and/or Effector Differentiation

#### Cytokines

##### IL-15, TGF-β, and IL-25

Numerous studies have demonstrated cytokines produced in the local environment play central roles in determining the differentiation of CD4^+^ T helper subsets (Th1, Th2, and Th17) (29). Similarly, the differentiation of iNKT subsets is heavily influenced by different cytokine signals (Figure 1 “Effector differentiation”). For instance, it’s been shown that NKT1 cells highly express CD122 (IL2Rβ), and CD122-mediated IL-15 signaling is essential for the differentiation of NKT1 cells (30). Likewise, the absence of TGF-β signaling (CD4-Cre × TGF-βRII^flox/flox^ and CD4-Cre × Smad4^flox/flox^) led to complete loss of ROR-t^+^ NKT17 cells (31). Both NKT2 and NKT17 cells express IL-17RB (IL-25 receptor), which was essential for the production of IL-13, IL-9, IL-10, and IL-17 after stimulation with αGalCer (13), demonstrating that the cytokine production of activated iNKT cells is influenced by a signal through this receptor. It was further shown that such effect was dependent on E4BP4, a transcription factor that regulates IL-10 and IL-13 production in CD4^+^ T and iNKT cells (32, 33). Interestingly, E4BP4 seems to be upregulated in iNKT cells only after stimulation with IL-25 or αGalCer (13, 33), but not expressed by thymic or most peripheral iNKT cells in the steady state (except the adipose iNKT cells) (13, 33, 34). Though inferred by the data, iNKT subsets defined by transcription factor expression as NKT1, 2, and 17 were not directly evaluated in the study (13). Thus, whether the development of NKT2 and/or NKT17 cells is controlled by the IL-17RB/IL-25 axis remains or be defined. In a scenario where IL-25 signaling controls differentiation of NKT cells, it would be important to define the source of IL-25 in the thymus (Figure 1; Table 1). A recent study demonstrated that a type of specialized epithelial cells, called tuft cells, are the solely source of IL-25 in the gut (35). It will be interesting to check the thymus for this lineage of epithelial cells as well.

#### Transcription Factors

##### Egr2

Strong TCR signaling in stage 0 iNKT cells commits their fate to iNKT lineage, as it leads to elevated expression of the transcription factors Egr1 and Egr2, which influence further development of iNKT cells (36). In agreement with Egr2 directly binding the PLZF promoter, Egr1 and Egr2 together are critical for PLZF induction, which indicates that Egr1 and Egr2 may be upstream of PLZF in determining iNKT lineage fate (36). In addition, Egr2-deficient iNKT cells failed to express CD122, indicating that elevated Egr2 expression not only specifies iNKT lineage at an early stage but its sustained expression may also further influence differentiation of iNKT subsets (36). In addition, a cytoskeletal remodeling protein, P21-activated kinase 2 (Pak2) also influences the development of iNKT cells, especially NKT1 and NKT2 cells, possibly through regulation of the two critical transcription factors, Egr2 and PLZF (37).

##### KLF Family Factors

The transcription factor Kruppel-like factor 2 (KLF2) is essential for T cells egress from thymus and lymph node, because it’s required for the expression of sphingosine 1 phosphate receptor type 1 (S1P1) in T cells (38). Unexpectedly, thymocytes in KLF2-deficient mice (CD4-Cre × KLF2^flox/flox^) displayed a memory phenotype (CD44^high^ CXCR3^+^ CD122^+^) that was shown to be an IL-4-dependent cell-nonautonomous effect (39). Furthermore, this effect was due to the expansion of IL-4-producing PLZF^high^ T cells (mostly NKT2 cells), showing that KLF2 negatively regulates the differentiation of NKT2 cells (40). Another member of the Kruppel-like family, KLF13, plays the opposite role. KLF13 deficiency (KLF13^−/−^) led to a diminished population of IL-4-producing PLZF^high^ iNKT cells (41).

##### Hobit

Though serving as an important factor that instructs the tissue retention program in iNKT cells and resident memory T cells (Trm) (42), the transcription factor Hobit was also shown to regulate the differentiation of iNKT cells (43). Hobit expression is high in CD44^high^ NK1.1^+^ iNKT cells (mostly NKT1 cells), but low in CD44^low^ NK1.1^−^ and CD44^high^ NK1.1^−^ iNKT cells (mostly NKT17 and NKT2 cells) (43). Accordingly, the number of CD44^high^ NK1.1^+^ iNKT cells was significantly reduced in Hobit-deficient mice, while the abundance of CD44^low^ NK1.1^−^ and CD44^high^ NK1.1^−^ iNKT cells remained intact (43). Though the iNKT subsets were not distinguished in the study, it could be inferred from the data that Hobit promotes the differentiation and/or thymic retention of NKT1 cells.

##### Lymphoid Enhancer Factor 1 (LEF1) and T Cell Factor 1 (TCF1)

The transcription factors LEF1 and TCF1 are essential for T cell development including early commitment to the T cell fate, transition from DN to the DP thymocytes, as well as following CD4/CD8 choice (44). The critical role of LEF1 and TCF1 in the differentiation of iNKT subsets has also been shown. Deletion of TCF1 at DP stage (CD4-Cre × Tcf7^flox/flox^) led to a severe defect in all three iNKT subsets (45). In addition, iNKT cell development was similarly impaired in absence of LEF1 (Vav-Cre × Lef1^flox/flox^) (46). LEF1 was required for the proliferation and survival of iNKT cells, especially the massive expansion after stage 0 (46). Interestingly, though it influenced the development of all three iNKT subsets, LEF1 showed a preference in promoting the differentiation of NKT2 cells (46).

#### Chromatin Modifiers

Epigenetic modifications also regulate development and differentiation of iNKT cells. The TET-family dioxygenases, TET1, TET2, and TET3, oxidize 5-methylcytosine (5mC) to 5-hydroxymethylcytosine (5hmC), which is an important DNA modification critical for various biological processes (47–49). Simultaneous deletion of *Tet2* and *Tet3* resulted in uncontrolled TCR-mediated expansion of NKT17 cells through suppression of T-bet and ThPOK (50). Jarid2, a component of polycomb repressive complex 2 that methylates histone 3 lysine 27 (H3K27), is also involved in iNKT cells development. Upregulated after TCR stimulation, Jarid2 directly binds to the PLZF promotor as a transcriptional repressor. Therefore, deficiency of Jarid2 led to significant expansion of PLZF^high^ NKT2 cells (51). In addition, the transcriptional repressor NKAP was shown to be required for the development of iNKT cells, as the iNKT development was completely abrogated at stage 0 in mice deficient of NKAP (CD4-Cre × NKAP^flox/flox^) (52). How NKAP regulates iNKT cell development is not clear, but its interaction with the histone deacetylase 3 (Hdac3) may be important, as NKAP is known to associate with Hdac3 and a similar defect of iNKT cells was observed in Hdac 3 conditional knockout mice (CD4-Cre × Hdac3^flox/flox^) (53). A recent report demonstrated that the H3K27me3 histone demethylase UTX is essential for iNKT cell development, especially the differentiation of NKT1 cells, as there was considerably fewer T-bet^+^ NKT1 cells in UTX-deficient mice while NKT2 and NKT17 cells were not affected (54). UTX not only directly binds to the promoters of T-bet and CD122 genes but also influences the epigenetic landscape and transcription of PLZF-activated genes (54).

#### MicroRNAs (miRNAs)

MicroRNAs are small noncoding single-strand RNAs (~22 nt) that modulate the stability and transcriptional activities of messenger RNAs (mRNAs) and *via* this mechanism influence the transcriptomes of various cells, leading to further effects on cellular proliferation, apoptosis, lineage commitment, and differentiation (55). Perhaps not surprisingly, complete loss of mature iNKT cells was observed in mice lacking Dicer (CD4-Cre × Dicer^flox/flox^), which are incapable of generating functional miRNAs in T cells, thus demonstrating that miRNAs are essential for the development of iNKT cells (56). miR-181a is abundant in DP thymocytes and could augment TCR signaling strength *via* enhancing the basal activation of TCR signaling molecules, such as increased basal phosphorylation level of Lck and ERK (57). Deletion of miR-181a (miR-181a/b-1^−/−^ mice) completely blocked iNKT cell development at the DP/Stage 0, which was presumably due to reduced responsiveness to TCR signals as exogenous agonistic ligand (αGalCer) could rescue iNKT cell generation (58). The miR-17–92 family cluster is also critical for the development of iNKT cells, in that absence of miRNAs of the miR-17–92 family cluster (triple knockout of three paralogs miR-17–92, miR-106a–363, and miR-106b–25 clusters) resulted in almost complete ablation of the three iNKT effector subsets (59). Excessive TGF-β signaling was seen in the remaining triple knockout iNKT cells, but it did not solely account for the impaired iNKT cell development, because deletion of TGF-βRII did not fully restore the hemostasis of iNKT cells (59). It was further found that the Let-7 family miRNAs, the most abundant family of miRNAs in mammals, tightly controls the differentiation of iNKT subsets (60, 61). Let-7 miRNAs are abundant in NKT1 cells while low in NKT2 and NKT17 cells, targeting *Zbtb46* mRNAs and inhibiting PLZF expression, therefore, directing iNKT cell differentiation into PLZF^low^ NKT1 lineage (61). Moreover, Lin28 inversely regulates Let-7 miRNAs, and Lin28 transgenic mice, which are practically deficient in Let-7 miRNAs, showed significantly increased NKT2 and NKT17 cells (61). miR-150 is expressed in lymphocytes (B, T, and NK cells) and has been implicated in their maturation. Correspondingly, miR-150 expression is expressed in iNKT cells after stage 0 (62, 63). In a mixed bone marrow chimera system, cell-intrinsic deficiency of miR-150 mildly affected iNKT cell development (62, 63), while overexpression of miR-150 substantially blocked maturation of iNKT cells beyond stage 0 (62). This suggests that fine-tuning of miR-150 level might be critical for iNKT cell development. Though the molecular pathway underlying this miR-150-dependent iNKT cell development is unclear, regulation of c-Myb by miR-150 could be involved (62, 63).

#### Cellular Protein Degradation System

While playing a central role in iNKT cell development, PLZF is initially induced in the stage 0 iNKT cells, and its expression can be regulated by the transcription factor Runx1 through direct binding to a critical enhancer of PLZF gene (64). Using Chip-Seq analysis, PLZF was shown to bind and regulate multiple genes, especially a broad set of immune effector genes expressed in iNKT cells (65). Beside directly regulating the expression of various genes, PLZF was also shown to transport an E3 ubiquitin ligase, cullin 3 (CUL3), from cytosol to nucleus, which would induce unique and essential ubiquitination patterns in iNKT cells (66). The number of iNKT cells was dramatically decreased in mice lacking CUL3 (CD4-Cre × CUL3^flox/flox^), further substantiating the importance of PLZF–CUL3 interaction in the development of iNKT cells (66). In line with its association with CUL3, PLZF has also been reported to interact with enhancer of zeste homolog 2 (Ezh2) methyltransferase (67). Moreover, Ezh2 directly methylates PLZF, causing its ubiquitination and subsequent degradation. Deletion of Ezh2 leads to sustained expression of PLZF and substantial expansion of PLZF^high^ NK1.1^−^ iNKT cells (mostly IL-4-producing NKT2 cells) (67).

#### Endogenous Selecting Lipid-Ligand and TCR Specificity

The generation of iNKT cells depends on recognition of lipid antigen presented by CD1d molecules on DP thymocytes. This antigen is most likely to be a self-lipid(s), because iNKT cells emerge early in life (6, 68), before stable colonization of commensal bacterial. Moreover, the phenotype and function of thymic and most peripheral iNKT cells (except pulmonary and intestinal iNKT cells) are normal in germ-free mice (69, 70). Regulated lipid metabolism in DP thymocytes is critical for thymic selection of iNKT cells, and the transcription factor Bcl11b plays a vital role in this process (71). Bcl11b-deficient (CD4-Cre × Bcl11b^flox/flox^) thymocytes showed deficient presentation of endogenous lipid antigens, dysregulated endo-lysosomal compartment, and alterations in genes involved in lipid metabolism (71). Moreover, in a mixed bone marrow chimera system, Bcl11b-deficient DP thymocytes (TCR-α^−/−^/CD4-Cre × Bcl11b^flox/flox^) failed to support selection of iNKT precursors in Bcl11b-sufficient DP thymocytes (β2m^−/−^/Bcl11b-Wt) (71). CD1d molecules can traffic between cell membrane and cytosolic organelles, surveying the endo-lysosomal compartment (72). A mouse model that expresses CD1d with a truncated cytoplasmic tail showed a severe defect in intracellular trafficking, and the number of iNKT cells was significantly reduced, suggesting the selection of iNKT cells relies on endosomal trafficking of CD1d molecule (73).

**Figure 2:**
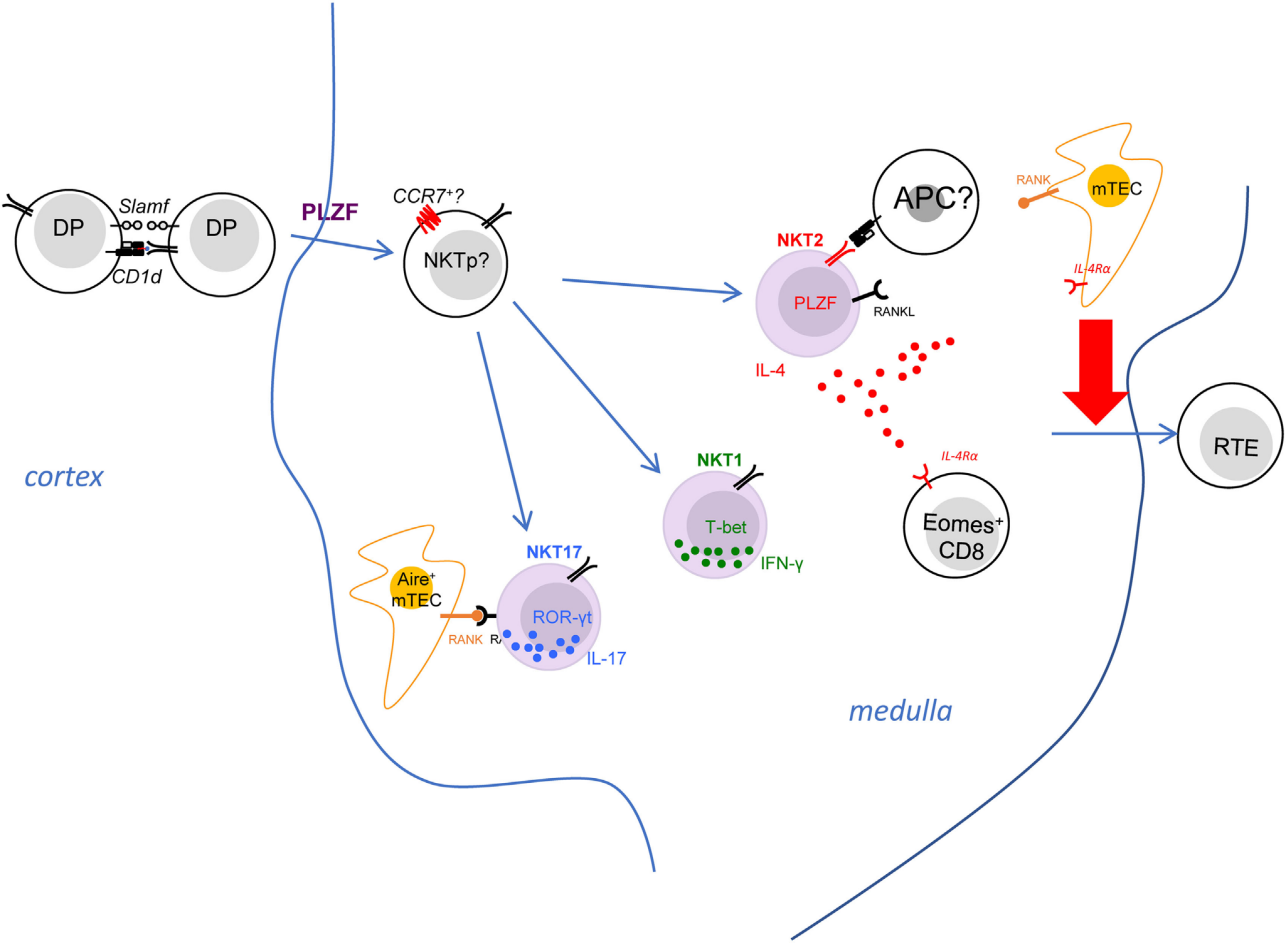
The invariant natural killer T (iNKT) effector subsets modulate immune homeostasis in the thymic medulla. The iNKT effector subsets predominantly reside in the thymic medullary area. Both NKT2 and NKT17 cells express RANK ligand, which interacts with RANK on medullary thymic epithelial (mTEC) to induce Aire expression. NKT2 cells also produce IL-4 at steady state, which has a striking effect on CD8^+^ single positive thymocytes—causing them to upregulate Eomes and adopt memory-like phenotype and function. The iNKT-derived type 2 cytokines, IL-4 and IL-13, also influence mTEC to promote emigration of mature thymocytes, through and as-yet undefined mechanism. The activation requirements for NKT2 cells are poorly defined, although they do need T cell receptor stimulation to produce IL-4 in the medulla.

Though a great effort has been made to understand the stimulatory thymic self lipid(s), controversy remains, as reviewed elsewhere (22, 74, 75). Briefly, iGb3, an endogenous lysosomal glycosphingolipid, though thought to be presented by LPS-activated dendritic cells that activate iNKT cells (76), is unlikely to be a major selecting ligand for iNKT cells given that the development and function of iNKT cells are normal in isoglobotrihexosylceramide (iGb3)-deficient mice (77). Instead, glycosphingolipids (GSL) have been implicated in the development of iNKT cells as mice deficient of GSL-synthesizing enzyme glucosylceramide (GlcCer) synthase (GSC) in hematopoietic cells (Vav-Cre × GCS^flox/flox^) showed mild reduction of iNKT cells in both thymus and periphery (78). While stage 0 iNKT cells were not examined in the study, it remains unclear whether GSL are involved in the positive selection of iNKT cells. A recent report demonstrated the selecting ligands likely to be α-linked glycosylceramides (79). Since all glycosylceramides in mammals were believed to be β-anomers due to that mammalian glycosylceramide synthases are β-transferases (80), this finding is somewhat surprising. Earlier studies pioneered by the Brenner group showed, though initially thought to be a potent lipid self-antigen for iNKT cells, that β-glucopyranosylceramide (β-GlcCer) actually does NOT possess antigenic activity to iNKT cells (81, 82). The observed activity of β-GlcCer is likely due to inclusion of an α-GlcCer species (82). These observations suggested the possibility that α-glycolipids are endogenous antigenic lipids for iNKT cells (82). However, nuclear magnetic resonance spectroscopy analysis at the time did not render a definitive identity (82). It is possible that an unknown alternative enzymatic pathway, unfaithful enzymatic activities, or unique stressed cellular environments could confer production of small amounts of α-linked glycolipids, though the exact mechanism remain to be discovered (79, 83). The peroxisome-derived ether lipids seem to be partially involved in the iNKT cell development, as mice deficient in the peroxisomal enzyme glyceronephosphate O-acyltransferase (GNPAT) harbor moderately reduced iNKT cells and GNPAT^−/−^ thymocytes are unable to support maturation of iNKT cells (84). However, the number of stage 0 iNKT cells are NOT changed in GNPAT^−/−^ mice (84), suggesting that peroxisome-derived lipids may not be the predominant selecting ligands for iNKT cells, but rather influence later developmental events of iNKT cells. The lysosomal phospholipase A2 (LPLA2), which modifies lysophospholipids in the lysosome, has been shown to play a role in thymic selection of iNKT cells, as both CD1d endogenous antigen presentation and iNKT cell numbers were negatively affected in the absence of LPLA2 (85). Taken together, considering that maturation of iNKT cells after positive selection of stage 0 iNKT cells requires the presence of CD1d in the thymus (86), it is possible that the endogenous lipid ligands for iNKT cells are presented in both thymic cortex and medulla and are displayed by different antigen-presenting cells (APCs). In this fashion, they may influence both selection (in the cortex) and effector differentiation (in the medulla) of iNKT cells (Figure 2).

Consistent with a potential role of specific self-lipids in effector differentiation, it was noted that the three iNKT subsets express distinct but stable Vβ repertoires (11, 87, 88). For example, NKT2 cells show a higher usage of Vβ7 (11). Thus, a few studies have raised the hypothesis that differential TCR signaling events due to biased TCR Vβ gene usage could impact the differentiation of iNKT subsets (87, 88). Through generation of retrogenic mice expressing different CDR3β to manipulate iNKT TCR β chain *in vivo*, a recent study clearly demonstrated the half-life of TCR-Ag-CD1d interaction governs the frequency of different iNKT subsets in a cell-intrinsic manner. The number of NKT2 cells strongly correlated with the *t*_1/2_ of tetramer binding (89). As mentioned above, a high level of Nur77GFP was seen in NKT2 cells in the steady state, suggesting continuous TCR signaling in NKT2 cells (11). However, it is less clear whether such continuous TCR stimulation is required for the steady-state production of IL-4 in NKT2 cells and/or the development of PLZF^high^ NKT2 cells (Figure 2). Since NKT2 cells reside in the thymic medulla, further efforts are required to elucidate where and how TCR binding kinetics of NKT2 cells might control their differentiation (Figure 2).

## iNKT Cells Modulate Tissue Homeostasis

### Major Role of iNKT-Derived IL-4

#### Thymus

Using KN2 mice, in which a human CD2 cassette was knocked into the IL-4 gene locus, and human CD2 expression on cell surface indicates active secretion of IL-4, a previous study demonstrated that thymic NKT2 cells produce abundant IL-4 at steady state (11). Another group showed thymic iNKT cells may also produce IL-13 at steady state in IL-13GFP mice (90). Because iNKT cells are predominantly localized in the medulla, IL-4 produced by NKT2 cells could influence a variety of immune events in that environment (Figure 2). Indeed, steady-state production of IL-4 selectively activates STAT6 in medullary CD8^+^ single-positive thymocytes, which drives them to become memory phenotype (CXCR3^+^ CD122^+^ Eomes^+^) (11, 40). This population of IL-4-induced memory T cells has been categorized as innate memory T cells (91), and they maintain greater function compared to naïve CD8^+^ T cells. They are well equipped to produce IFN-γ in response to TCR stimulation and showed much better expansion after infection with *listeria monocytes* (LM) (40). Moreover, the developmental exposure to IL-4 is critical for CD8^+^ T cells to mount robust Th1 responses to acute or chronic lymphocytic choriomeningitis virus infection (92, 93). Therefore, innate memory T cells are beneficial to the host for their functional superiority (94). Nevertheless, we do not yet understand, in the bigger picture, why iNKT cells recognition of medullary self-lipids should control this process.

IL-4 impacts other immune cells beyond CD8 T cells in thymic medulla. A recent study demonstrated that the type 2 cytokines (IL-4/13) produced by iNKT cells could influence in the thymic emigration of mature thymocytes (90). IL-4Rα^−/−^ mice showed accumulation of mature T cell in the thymus and reduced recent thymic emigrants in the periphery (90). Medullary thymic epithelial (mTEC) cells express IL-4Rα and can respond to the type 2 cytokines, as pSTAT6 level went up in mTEC of FTOC when IL-4 and IL-13 were added in the culture (90). Moreover, disorganization of the thymic medulla was observed in mice deficient of IL-4Rα, that the medulla contained some epithelial-free areas revealed by the ERTR5 staining (90). It was speculated that IL-4/13 signaling in mTEC might promote the egress of mature T cells from thymus, though the specific mechanism remains to be uncovered (90). While the S1p–S1p1 axis remains intact in the IL-4Rα^−/−^ mice (90), it is possible that the IL-4/13 produced by NKT2 cells serve as a novel regulator of thymic emigration of T cells.

#### Periphery

In the periphery, iNKT cells are critical for restoring homeostasis under stressed conditions. The regulatory role of iNKT cells has been implicated in the type 1 diabetes, where iNKT cells are less frequent and biased toward Th1 cytokine production in diabetic siblings than in their non-diabetic siblings (95). The protective role of iNKT cells has been shown in the mouse model for type 1 diabetes [non-obese diabetes (NOD) mouse], as CD1d^−/−^ NOD mice, lacking iNKT cells, have a higher risk and earlier onsets of diabetes compared to CD1d^+/+^ counterparts (96). Such protection is dependent on the IL-4 production by iNKT cells (97, 98), and activation of iNKT cells to produce IL-4 by cognate lipid antigen α-Galcer prevents diabetes in NOD mice (99, 100).

Recent studies highlighted the key role of iNKT cells in regulating the pathogenesis of graft-versus-host disease (GvHD), a severe immunological dysregulation that frequently occurs after allogeneic hematopoietic stem cell transplantation (101, 102). Higher frequency of iNKT cells in patients correlated with lower risk of GvHD (102). In murine studies, stimulation with α-Galcer or adoptive transfer of iNKT cells confer substantial protection against GvHD (103, 104). Furthermore, the iNKT cell-derived IL-4 and following regulatory T cells expansion seem to be critical for optimal suppression of GvHD (102–104). These data point iNKT cells as promising therapeutic regimen for GvHD patients.

Invariant natural killer T cells are rare in most peripheral sites (0.1–1% of lymphocytes), but highly enriched in the liver, representing nearly 30% of hepatic lymphocytes (23). They are actively involved in restoring tissue homeostasis after sterile liver injury as demonstrated in a recent report (105). Shown by intravital microscopy, iNKT cells randomly patrol the sinusoids within liver in the steady state, while they rapidly move toward the injury site after injury (105, 106). Arrested at the injury site due to TCR stimulation and IL-12/18 signals, iNKT cells produce IL-4 to promote a series of events that are vital for optimal tissue repair, including increased proliferation of hepatocytes, the switch of monocyte subtypes from CCR2^high^ CX3CR1^low^ to CCR2^low^ CX3CR1^high^, as well as reduced collagen deposition (105).

Altogether, these studies demonstrated that iNKT cells are potent regulator in immunity, largely due to their ability to produce abundant cytokines. Most of the studies implicated iNKT cell-derived IL-4 as the critical factor in restoring tissue homeostasis. Therefore, to unleash the therapeutic potential of iNKT cells, it will be important to have better understanding of the underlying mechanisms, especially the relevant APCs and the precise stimulatory lipid antigens that activate iNKT cells to produce IL-4.

### Role of Other iNKT Subsets

Invariant natural killer T cells also have strong potential to produce other cytokines (IFNγ by NKT1 and IL-17 by NKT17). However, the role of these subsets and cytokines on tissue homeostasis has not been deeply explored, although it should be noted that NKT17 are abundant in the lung. iNKT cells also express a variety of other stimulatory or inhibitory molecules; therefore, they might influence immune homeostasis through direct cell contact. One of the molecules expressed by iNKT cells is RANK ligand (RANKL) (24). Signals through tumor necrosis factor family receptors (TNFRSF) RANK promotes Aire expression in mTEC (107). While iNKT cells express RANKL, and Aire^+^ mTECs were significantly reduced in CD1d^−/−^ mice (24). It strongly suggests that iNKT cells could regulate the development of mTEC through direct cross-talk to induce RANK signals. Further RNA-Seq analysis demonstrates that only NKT2 and NKT17 cells highly express RANKL (28), suggesting that iNKT subsets may have unique effects in modulating tissue homeostasis in the thymus (Figure 2).

## The Parallels in Development of iNKT Cells and Mucosal-Associated Invariant T (MAIT) Cells

**Figure 3:**
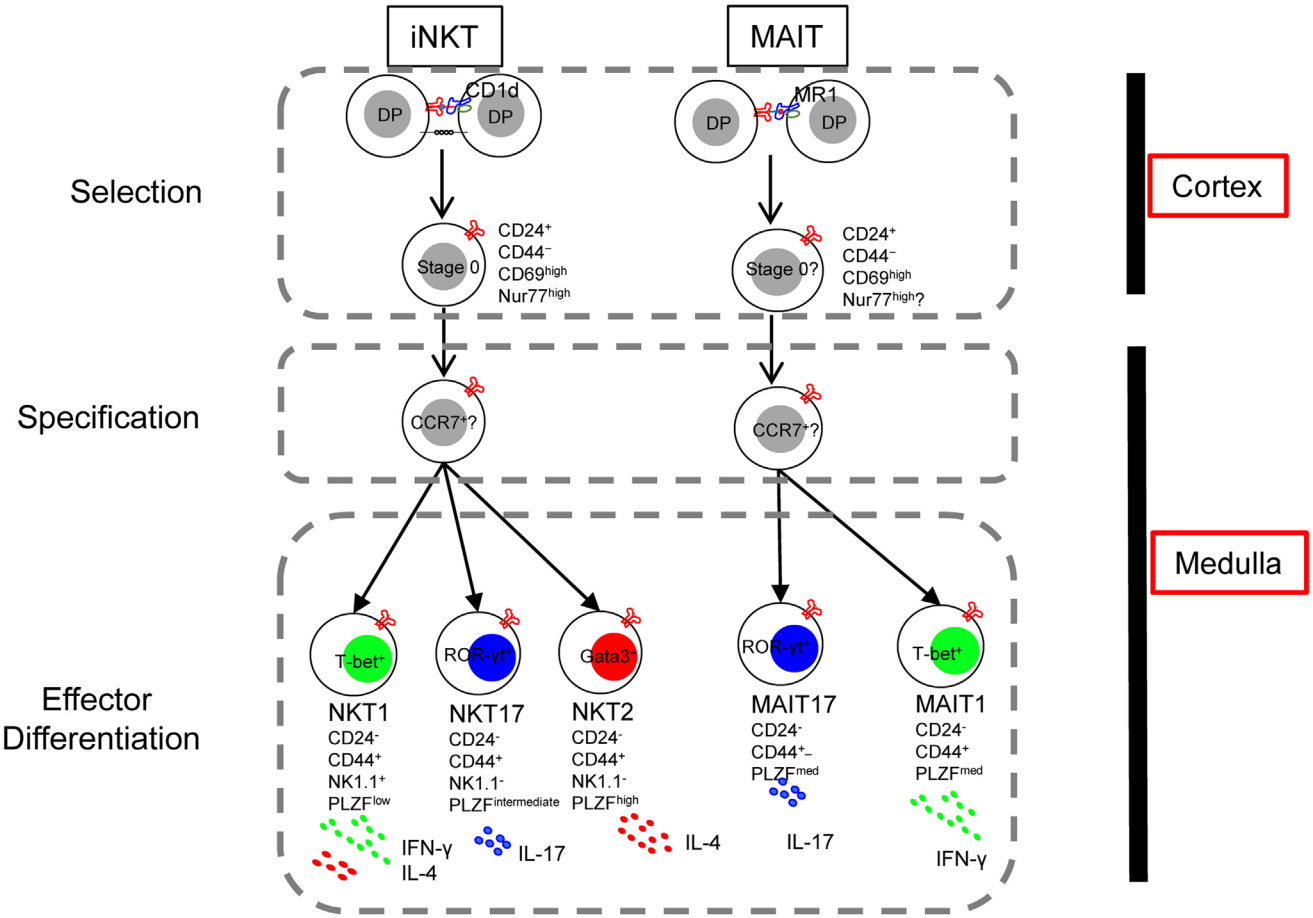
The lineage-diversification model of invariant natural killer T (iNKT) cell development parallels that of mucosal-associated invariant T (MAIT) cells. Interactions between double-positive (DP) thymocytes expressing the appropriate T cell receptor and CD1d or MR1 on neighboring thymocytes generates CD24^+^ CD44^−^ lineage committed stage 0 cells (Selection). With downregulation of CD24 and upregulation of the signature transcription factor PLZF, PLZF^high^ progenitor cells migrate from cortex to medulla, likely mediated by the chemokine receptor CCR7 (Specification). In the medulla, responding to various cues, the PLZF^high^ CCR7^+^ progenitor further differentiates into distinct effector subsets (Effector differentiation). Thus, the thymic development of iNKT cells and MAIT cells are parallel in many aspects except that an IL-4/13-producing MAIT cell has not been described.

The MAIT cells are another specialized lineage of innate-like T cells, expressing a semi-invariant TCR, that Vα7.2-Jα33 chain predominantly paired with Vβ2 or Vβ13 in human and Vα19-Jα33 chain predominantly paired with a Vβ6 or Vβ8 in mice (108). They are remarkably abundant in human tissues, making of 1–10% of T cells in peripheral blood, nearly 10% of T cells in intestine and up to 40% of T cells in liver (109, 110). Therefore, MAIT cells have attracted great interest in terms of elucidating their development and function. Recently, with the discovery of the vitamin B metabolites as cognate antigens and successful manufacturing of MR-1 tetramer to accurately detect MAIT cells in mice and human (111, 112), we have gained a more clear understanding of their development and homeostasis. Surprisingly, the thymic development of MAIT cells parallels many aspects of iNKT cells (Figure 3).

Mucosal-associated invariant T cells originate in the thymus where their selection depends on the interaction with the MR-1 expressing DP thymocytes (113). Positively selected immature MAIT cells are CD24^+^ CD44^−^, which give rise to the CD24^−^ CD44^+^ mature MAIT cells (70). These CD44^+^ MAIT cells are comprised of at least two distinct subsets, T-bet^+^ MAIT cells and ROR-γt^+^ MAIT cells reminiscences the NKT1 and NKT17 cells. Moreover, like iNKT cells, MAIT cells express PLZF and depends on PLZF for their differentiation, as CD44^+^ MAIT cells were absent in PLZF-null mice (70). Furthermore, microRNA plays indispensable role in the development of both MAIT cells and iNKT cells, the expansion and differentiation of MAIT cells beyond the CD24^+^ stage were severely impaired in Drosha-deficient mice (70). With the notion that MAIT cells development might parallel the development of iNKT cells, it is reasonable to reference what we learned from iNKT cells to facilitate and advance our understanding of MAIT cells. Many tools designed and hypotheses raised for research of iNKT cells could be applied to that of MAIT cells. Using CD24 and CD44 to distinguish immature and mature MAIT cells, as well as examine expression and dependency of PLZF in MAIT cells are both good examples of that. Taken one step further, more questions could be asked: (1) whether MAIT cells receive strong TCR signal like iNKT cells during selection; (2) whether the two MAIT cell effector subsets require differentiation cues similar to those for NKT1 and NKT17 cells; (3) whether thymic MAIT effector cells predominantly reside in medulla; (4) and PLZF induce tissue residency program in iNKT cells—is it the same in MAIT cells?

## More iNKT Subsets: NKT10, NKT_FH_, and Adipose iNKT

Beside the three effector subsets in the thymus, additional functional subpopulations of iNKT cells have been described. Follicular helper iNKT cells (NKT_FH_) were detected after immunization with α-GalCer-conjugated proteins or haptens (114, 115). NKT_FH_ adopt the phenotype of MHC-II restricted T follicular helper cells (T_FH_), expressing a variety of classical T_FH_ surface markers and transcription factor, including PD-1, CXCR5, ICOS, and Bcl6 (114, 115). NKT_FH_ initiate and localize in germinal centers, provide both cognate and noncognate help to lipid and protein-specific B cells, respectively (114, 115). However, NKT_FH_-dependent germinal center reactions failed to generate long-lived plasma cells (114). Another specialized subpopulation of iNKT cells emerges after stimulation with αGalCer is the regulatory NKT10 cells, characterized by predominant IL-10 production (33). Unlike T regulatory (T_reg_) cells, NKT10 don’t express Foxp3, rather, they highly express E4BP4, a transcription factor regulates IL-10 and IL-13 production in CD4 T and iNKT cells (32, 33).

Adipose iNKT cells have gained focus for their crucial role in modulating T_reg_ cells and macrophages, which are correlated with the onset of obesity (116). However, the generation or selection of adipose iNKT cells has been a puzzle. Interestingly, adipose iNKT cells have been found to share phenotype with NKT10 cells, in that they both produce abundant IL-10 and rely on E4BP4 for their regulatory function (34). A recent discovery showed that recognition of CD1d by iNKT TCR controls the development of iNKT cells in the adipose tissue (117). TCRα–TCRβ pairing of iNKT TCR creates a hydrophobic patch, which is critical for maintaining TCR conformation as well as its recognition of CD1d molecule (117). Partial disruption of this patch by substitution of a single amino acid in TCR Vβ8.2 chain (F108Y), while recognition of CD1d preserved, significantly alters the development of iNKT cells, results in an enrichment of iNKT cells in the adipose tissue (117). It is unclear whether this is due to altered selection in the thymus or enhanced proliferation/competitive advantage of adipose iNKT cells on site.

## Concluding Remarks

T cells play a central role in protecting the body from infectious agents and cancer, but at the same time can cause autoimmune diseases when dysregulated. iNKT cells are a specialized lineage of T cells that recognize foreign and self-lipids in a manner quite distinct from conventional T cells. Though iNKT cells are a numerically small population, their striking ability to rapidly produce large amounts of cytokines renders them potent regulators of immunity—implicated in antimicrobial responses, antitumor immunity, and autoimmune and allergic diseases. Despite past progress, a number of questions regarding the development of iNKT cells remain unanswered. First, what is the nature of endogenous lipids recognized by iNKT cells, especially the lipids presented by cortical DP thymocytes that induce positive selection of iNKT cells? Second, evidence suggests that NKT2 cells produce large amounts of IL-4 at steady state in the thymus. It is of great interest to understand how this process is regulated. Are antigenic lipids and TCR stimulation required, and if so what is the identity of APCs?

Finally, though iNKT cells are found in most tissues, the frequency of iNKT subsets varies greatly in different organs. For instance, NKT17 are enriched in lung and skin draining LN, while liver iNKT cells are predominantly NKT1. What dictates this striking bias in the distribution of iNKT subsets? How does this differential distribution influence immune responses and/or modulate tissue homeostasis? What is the phenotype of iNKT cells that recently emigrated from thymus to seed in periphery? What are the environmental and cell-intrinsic factors that regulate differentiation or homing of iNKT subsets in various peripheral sites? iNKT stimulatory lipids are well-tolerated in human trials. Through selective activation of different iNKT effector subsets, iNKT cells can modulate immune responses and tissue homeostasis in different fashions. This can only be possible with a better understanding of the developmental steps that drive iNKT cells into functionally distinct subsets.

## Author Contributions

HW drafted the manuscript. KH supervised the writing and edited the manuscript.

## Conflict of Interest Statement

The authors declare that the research was conducted in the absence of any commercial or financial relationships that could be construed as a potential conflict of interest. The reviewer SJ and handling Editor declared their shared affiliation.
